# 单中心906例真性红细胞增多症患者生存预后因素分析

**DOI:** 10.3760/cma.j.issn.0253-2727.2021.11.003

**Published:** 2021-11

**Authors:** 丹 刘, 泽锋 徐, 培红 张, 娇 马, 铁军 秦, 士强 曲, 秀娟 孙, 冰 李, 丽娟 潘, 玉娇 贾, 志坚 肖

**Affiliations:** 中国医学科学院血液病医院（中国医学科学院血液学研究所），实验血液学国家重点实验室，国家血液系统疾病临床医学研究中心，天津 300020 State Key Laboratory of Experimental Hematology, National Clinical Research Center for Blood Diseases, Institute of Hematology & Blood Diseases Hospital, Chinese Academy of Medical Science & Peking Union Medical College, Tianjin 300020, China

**Keywords:** 真性红细胞增多症, 总生存, 血小板分布宽度, 预后, Polycythemia vera, Overall survival, Platelet distribution width, Prognosis

## Abstract

**目的:**

探讨影响真性红细胞增多症（PV）患者总生存（OS）的预后因素。

**方法:**

对2007年6月至2020年2月就诊于中国医学科学院血液病医院的906例符合WHO（2016）诊断分型标准的初诊PV患者进行回顾性分析。

**结果:**

906例患者中，男439例（48.5％），女467例（51.5％），中位确诊年龄为57（18～91）岁。31.6％（276/874）的患者有血栓史，27.2％（232/852）的患者左肋缘下可触及脾脏，4.6％（25/541）的患者伴有克隆性染色体核型异常。中位随访时间为54（95％*CI* 8～130）个月，5、10年累积死亡率分别为5.8％（95％ *CI* 4.8％～6.7％）、11.1％（95％*CI* 9.3％～12.9％）。单因素分析显示，年龄≥60岁、既往有血栓史、WBC≥15×10^9^/L、PLT≥450×10^9^/L、血小板分布宽度（PDW）≥15 fl是OS的危险因素，脾脏左肋缘下可触及是OS的保护因素。Cox回归多因素分析显示，年龄≥60岁（*HR*＝4.3，95％*CI* 2.1～9.2，*P*<0.001）、PDW≥15 fl（*HR*＝2.1，95％*CI* 1.1～4.0，*P*＝0.023）是OS的独立危险因素。PDW≥15 fl、<15 fl患者5年累积死亡率分别为8.6％（95％*CI* 5.9％～11.3％）、4.4％（95％*CI* 3.4％～5.4％）。国际工作组PV预后积分系统（IWG-PV）低危、中危、高危组患者5年累积死亡率分别为0.8％（95％*CI* 0.2％～1.4％）、4.0％（95％*CI* 2.7％～5.3％）、12.0％（95％*CI* 9.6％～14.4％）（*P*<0.05）。PDW对IWG-PV中危、高危患者的OS具有显著影响（*HR*＝2.3，95％*CI* 1.2～4.2，*P*＝0.009），而对低危患者没有影响（*HR*＝3.1，95％*CI* 0.2～52.0，*P*＝0.405）。

**结论:**

年龄≥60岁和PDW≥15 fl是影响中国PV患者OS的独立危险因素。IWG-PV预后积分系统适用于中国PV患者生存预测。

## Abstract (en)

**Objective:**

To explore predictors of overall survival (OS) in Chinese patients with polycythemia vera (PV).

**Methods:**

A total of 906 consecutive newly diagnosed patients with PV seen at the Blood Diseases Hospital, Chinese Academy of Medical Sciences, from June 2007 to February 2020 were included, and their data were collected. PV was diagnosed according to 2016 World Health Organization (WHO) diagnostic definitions. OS and prognostic factors were retrospectively analyzed.

**Results:**

Among the 906 patients, 439 were male (48.5％) and 467 were female (51.5％). The median age was 57 years (range: 18–91 years). 31.6％ (276/874) of the patients had a thrombosis history at diagnosis, and 4.6％ (25/541) of the patients had abnormal cytogenetics. The median follow-up was 54 months (95％ confidence interval [*CI*] 8–130 months). The 5- and 10-year cumulative deaths were 5.8％ (95％ *CI* 4.8％–6.7％) and 11.1％ (95％ *CI* 9.3％–12.9％）, respectively. Univariate analysis showed that age ≥60 years, thrombosis history, white blood cells (WBC) ≥15×10^9^/L, platelet (PLT) ≥450×10^9^/L, and platelet distribution width (PDW) ≥15 fl significantly correlated with worse OS, and palpable spleen correlated with better OS. Multivariate analysis showed that age ≥60 years (*HR*＝4.3, 95％ *CI* 2.1–9.2, *P*<0.001) and PDW ≥15 fl (*HR*＝2.1, 95％ *CI* 1.1–4.0, *P*＝0.023) were independent prognostic factors for worse OS. The 5-year cumulative death for patients with PDW ≥15 fl or PDW<15 fl was 8.6％ (95％ *CI* 5.9％–11.3％) or 4.4％ (95％ *CI* 3.4％–5.4％), respectively. The 5-year cumulative death for patients defined as low-, intermediate-, and high-risk patients by international working group score system for PV (IWG-PV) were 0.8％ (95 *CI* 0.2％–1.4％), 4.0％ (95％ *CI* 2.7％–5.3％), and 12％ (95％ *CI* 9.6％–14.4％), respectively, with a significant difference among the three cohorts (*P*<0.05). PDW ≥ 15 fl significantly affected OS for intermediate- and high-risk patients (*HR*＝2.3, 95％ *CI* 1.2–4.2, *P*＝0.009) defined by IWG-PV score system, but not for low-risk patients (*HR*＝3.1, 95％ *CI* 0.2–52.0, *P*＝0.405).

**Conclusion:**

Age ≥60 years and PDW ≥15 fl were independent prognostic factors for worse OS in PV. IWG-PV score system effectively predicted OS for Chinese patients with PV.

真性红细胞增多症（PV）是经典费城染色体阴性的骨髓增殖性肿瘤（MPN）之一，其特点为骨髓造血干细胞克隆性增殖引起外周血细胞增多[1]。PV患者中位生存期约为14年，显著短于性别、年龄匹配的健康人群[2]。既往研究发现年龄、白细胞计数（WBC）、血小板计数（PLT）、血栓史、染色体核型异常，以及SRSF2、ASXL1和IDH2基因突变是影响PV患者总生存（OS）的预后因素[3]–[5]。我们对2007年6月至2020年2月中国医学科学院血液病医院新诊断的906例PV患者进行回顾性分析，探讨PV患者的OS及其预后影响因素。

## 病例与方法

1. 病例：纳入本研究的病例为2007年6月至2020年2月在中国医学科学院血液病医院新诊断的PV患者。纳入标准包括：①符合世界卫生组织（WHO）2016版诊断分型标准[6]；②确诊时临床及实验室检查数据完整；③年龄≥18岁。

2. 染色体核型分析及二代测序（NGS）检测：染色体核型分析结果根据《人类细胞遗传学国际命名体制（ISCN2013）》的规定[7]进行描述，测序方法参考本中心此前报道[8]，检测基因组套涵盖112个血液肿瘤相关基因，用PCR引物扩增目的基因组，富集后采用Ion Torrent半导体测序平台进行测序[8]。平均基因覆盖率98.1％，平均测序深度1310×。

3. 治疗方案：所有患者口服阿司匹林100 mg/d预防血栓。66例（7.4％）患者未接受降细胞治疗，455例（50.2％）患者接受羟基脲（HU）治疗，155例（17.1％）接受重组干扰素（IFN）治疗，213例（23.5％）接受HU联合重组IFN治疗，2例（0.2％）接受芦可替尼治疗，15例死亡患者治疗方案不详。HU起始剂量为20 mg·kg^−1^·d^−1^，重组IFN起始剂量为300万单位（IU），每周3次。治疗方案由医师根据患者外周血细胞计数情况进行调整。

4. PV生存预后积分系统：采用国际工作组PV生存预后积分系统（IWG-PV）[3]：根据患者年龄（≥67岁赋值5分，57～66岁赋值2分）、WBC≥15×10^9^/L（1分）、静脉血栓史（1分），分为低危组（0分）、中危组（1～2分）和高危组（≥3分）。

5. 随访：随访截止日期为2020年10月30日，随访资料来源于住院、门诊病历及电话随访。OS定义为确诊日期至死亡日期或最后随访日期。

6. 统计学处理：偏态分布计量资料以中位数表示，组间比较采用非参数Mann-Whitney *U*检验。率的比较采用卡方检验或Fisher确切概率法。生存分析采用Kaplan-Meier法，生存曲线比较采用Log-rank检验，多因素分析采用Cox回归模型。生存分析连续变量的分组界值依据参考文献或应用X-tile软件确定。单因素分析*P*<0.1的影响因素纳入多因素分析。*P*<0.05认为差异具有统计学意义。所有数据采用SPSS 26.0软件包进行统计处理。

## 结果

1. 患者入组时临床和实验室检查特征：共906例患者纳入本研究，男439例（48.5％），女467例（51.5％），中位年龄为57（18～91）岁，43.2％（391/906）的患者年龄≥60岁。31.6％（276/874）的患者有血栓史，其中30.2％（264/874）有动脉血栓史，2.3％（20/874）有静脉血栓史。27.2％（232/852）的患者脾脏左肋缘下可触及，脾脏肿大患者脾脏左肋缘下中位4.0（1.0～16.5）cm。按PV血栓风险分组[9]，高危组占57.3％（501/874）（年龄≥60岁/有血栓史），低危组占42.6％（373/874）（年龄<60岁且没有血栓史）。根据IWG-PV生存预后积分系统[3]进行分组，低危组292例（33.3％），中危组291例（33.2％），高危组293例（32.3％）。

初诊血常规（中位数）：HGB 195（147～254）g/L，RBC 7.09（4.11～10.71）×10^12^/L，WBC 12.68（2.67～50.90）×10^9^/L，PLT 445（61～2169）×10^9^/L，红细胞比容（HCT）59.7％（45.4％～78.3％）。34.4％（312/906）的患者WBC≥15×10^9^/L，49.0％（444/906）的患者PLT≥450×10^9^/L。外周血涂片可见不成熟粒细胞（原始、幼稚粒细胞）或有核红细胞的患者比例分别为8.9％（72/806）、1.1％（9/806）。89.8％（316/352）的患者促红细胞生成素（EPO）降低（参考值2.6～18.5 IU/L）。

696例（76.8％）的患者进行了染色体核型分析，其中541例染色体核型结果可供分析。4.6％（25/541）的患者伴有克隆性染色体核型异常，9号染色体异常（8例）最为常见，其中伴有+9患者4例。1、2种染色体异常患者分别为20、4例，复杂染色体异常1例。843例（93.0％）患者检出JZK2V617F突变，21例（2.3％）检出JZK2 exon12突变，2例（0.2％）检出JZK2V617F、JAK2 exon12突变并存。JAK2V617F突变负荷（VAF）中位数为56％（95％*CI* 13％～87％）。

99例患者进行了二代测序基因突变检测，47例（47.5％）患者检出JAK2以外的其他基因突变，最常见的突变基因包括TET（18例，18.2％）和DNMT3A（8例，8.1％）。2例（2.0％）患者检出ASXL1突变，所有99例患者均未检测到既往报道与OS相关的SRSF2和IDH2基因突变[4]–[5]。

2. 生存分析：患者中位随访时间54（95％*CI* 8～130）个月。随访期间55例患者死亡，5年、10年累积死亡率分别为5.8％（95％ *CI* 4.8％～6.7％）、11.1％（95％*CI* 9.3％～12.9％）。死亡原因包括出血11例（20.0％）、血栓9例（16.4％）、感染6例（10.9％）、第二肿瘤5例（9.1％）、疾病进展3例（5.5％）、其他原因8例（14.5％）、死因不详13例（23.6％）。

3. 生存影响因素分析：依据文献[3]–[5]报道的PV患者OS影响因素，将年龄、血栓史、白细胞计数、血小板计数、异常染色体核型、外周血涂片可见不成熟粒细胞、ASXL1突变纳入生存分析。此外，我们还分析了血小板分布宽度（PDW）、JAK2突变类型、JAK2V617F VAF、促红细胞生成素（EPO）水平、外周血涂片可见有核红细胞、脾脏左肋缘下可触及、高血压病史、糖尿病史、冠心病史、降细胞治疗（HU、IFN或芦可替尼）对生存的影响。

（1）单因素分析：单因素分析显示，年龄≥60岁（*HR*＝5.4，95％*CI* 2.7～10.1，*P*<0.001）、血栓史（*HR*＝1.8，95％*CI* 1.0～3.1，*P*＝0.036）、WBC≥15×10^9^/L（*HR*＝1.9，95％*CI* 1.1～3.1，*P*＝0.020）、PLT≥450×10^9^/L（*HR*＝1.8，95％*CI* 1.1～3.1，*P*＝0.030）和PDW≥15 fl（*HR*＝2.0，95％*CI* 1.1～3.6，*P*＝0.022）是OS的危险因素，而脾脏左肋缘下可触及（*HR*＝0.4，95％*CI* 0.2～0.9，*P*＝0.028）是OS的保护因素，外周血涂片可见不成熟粒细胞或有核红细胞、JAK2突变类型、JAK2V617F VAF≥50％、异常染色体核型、ASXL1突变、EPO减低、降细胞治疗等对OS影响差异均无统计学意义（*P*值均>0.05）（表1）。单纯JAK2V617F突变与单纯JAK2 exon12突变患者比较，5年累积死亡率差异也没有统计学意义［5.9％（95％ *CI* 4.9％～6.9％）对0％，*P*＝0.370］。

（2）IWG-PV生存预后积分系统OS分组分析：按IWG-PV生存预后积分系统[3]分组，低危、中危、高危组患者中位随访时间分别为59（95％*CI* 3～133）、58（95％*CI* 7～137）、59（95％*CI* 12～32）个月，5年累积死亡率分别为0.8％（95％*CI* 0.2％～1.4％）、4.0％（95％*CI* 2.7％～5.3％）、12.0％（95％*CI* 9.6％～14.4％），差异具有统计学意义（*P*<0.001）（图1）。

（3）多因素分析：将单因素分析*P*值<0.1的影响因子纳入多因素分析。结果显示，年龄≥60岁（*HR*＝4.3，95％*CI* 2.1～9.2，*P*<0.001）和PDW≥15 fl（*HR*＝2.1，95％*CI* 1.1～4.0，*P*＝0.023）是影响OS的独立危险因素（表1、图2、图3）。年龄<60岁、≥60岁组5年累积死亡率分别为2.6％（95％*CI* 1.8％～3.4％）、10.2％（95％*CI* 8.3％～12.1％）。PDW<15 fl、≥15 fl患者5年累积死亡率分别为4.4％（95％*CI* 3.4％～5.4％）、8.6％（95％*CI* 5.9％～11.3％）。将PDW≥15 fl与IWG-PV预后分组纳入多因素分析，结果提示PDW≥15 fl是独立于IWG-PV生存预后分层的危险因素（*HR*＝2.0，95％*CI* 1.1～3.7，*P*＝0.026）。PDW≥15 fl对IWG-PV生存预后分层的中危和高危患者的OS具有显著影响（*HR*＝2.3，95％*CI* 1.2～4.2，*P*＝0.009）（图4），而对低危患者没有显著影响（*HR*＝3.1，95％*CI* 0.2～52.0，*P*＝0.405）。

**表1:** 影响真性红细胞增多症患者总生存的单因素和多因素分析

指标	单因素分析				多因素分析			
	纳入例数	*HR*	95％ *CI*	*P*值	纳入例数	*HR*	95％ *CI*	*P*值
年龄≥60岁	906	5.4	2.7～10.1	<0.001	727	4.3	2.1～9.2	<0.001
男性	906	0.9	0.5～1.5	0.594				
血栓史	874	1.8	1.0～3.1	0.036	727	1.5	0.8～2.8	0.209
静脉血栓史	874	0.8	0.2～9.5	0.789				
脾脏左肋缘下可触及	852	0.4	0.2～0.9	0.028	727	0.5	0.2～1.1	0.086
WBC≥15×10^9^/L	906	1.9	1.1～3.1	0.020	727	1.8	1.0～3.5	0.062
PLT≥450×10^9^/L	906	1.8	1.1～3.1	0.030	727	1.3	0.7～2.5	0.445
PDW≥15 fl	795	2.0	1.1～3.6	0.022	727	2.1	1.1～4.0	0.023
外周血涂片可见不成熟粒细胞	806	1.8	0.9～3.9	0.116				
外周血涂片可见有核红细胞	806	2.2	0.3～15.7	0.437				
JAK2 exon12突变	906	0.5	0～850.9	0.361				
JAK2V617F突变	906	1.9	0.5～7.9	0.351				
JAK2V617F突变负荷≥50％	657	0.9	0.5～1.8	0.857				
EPO减低	352	1.1	0.4～3.0	0.918				
异常染色体核型	541	2.3	0.7～7.8	0.158				
ASXL1突变	99	0.05	0～6.0	0.838				
高血压病史	754	1.3	0.7～2.5	0.409				
冠心病病史	754	1.0	0.3～3.1	0.942				
糖尿病病史	754	1.5	0.5～4.1	0.468				
降细胞治疗	891	0.5	0.2～1.4	0.171				

注：PDW：血小板分布宽度；EPO：促红细胞生成素

**图1:**
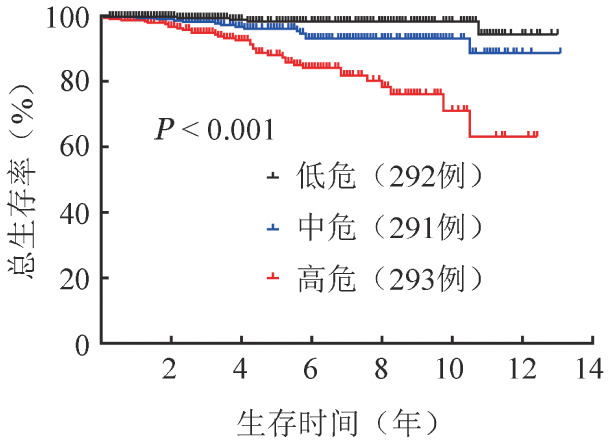
不同IWG-PV预后分组真性红细胞增多症患者总生存曲线 IWG-PV：国际工作组真性红细胞增多症生存预后积分系统

**图2:**
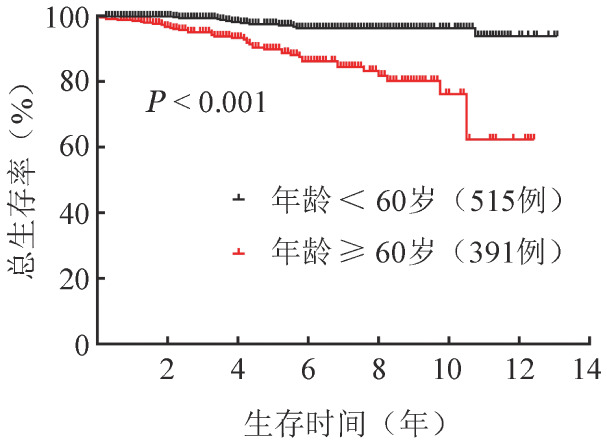
年龄≥60岁、<60岁真性红细胞增多症患者总生存曲线

**图3:**
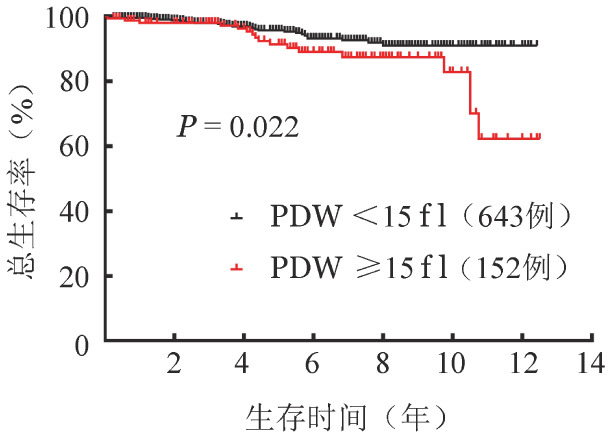
血小板分布宽度（PDW）对真性红细胞增多症患者总生存的影响

**图4:**
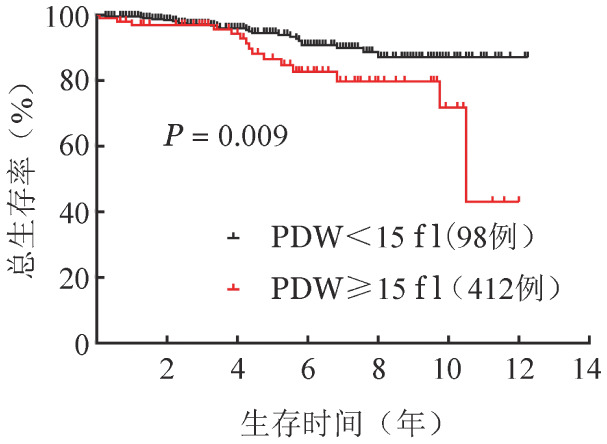
血小板分布宽度（PDW）对IWG-PV中、高危真性红细胞增多症患者总生存的影响 IWG-PV：国际工作组真性红细胞增多症生存预后积分系统

4. PDW影响OS的可能相关因素分析：比较PDW≥15 fl与PDW<15 fl患者的临床特征，发现PDW≥15 fl组女性占比较高（59.2％对48.7％，*P*＝0.019），脾脏左肋缘下可触及的患者占比较高（33.8％对24.3％，*P*＝0.019），具有更高的中位HGB（200 g/L对195 g/L，*P*＝0.003）、RBC（7.42×10^12^/L对6.95×10^12^/L，*P*<0.001）、HCT（62.2％对58.9％，*P*<0.001）和JAK2V617F突变负荷（64％对53％，*P*<0.001），两组年龄差异没有统计学意义（57岁对58岁，*P*＝0.392）（表2）。

**表2:** 血小板分布宽度（PDW）<15 fl与≥15 fl组真性红细胞增多症患者临床特征比较

临床特征	例数	PDW<15 fl组（643例）	PDW≥15 fl组（152例）	统计量	*P*值
年龄［岁，*M*（范围）］	795	58（18～84）	57（25～86）	*z*＝−0.857	0.392
年龄≥60岁［例（％）］	795	279（43.4）	66（43.4）	*χ*^2^＝0.000	0.995
女性占比［例（％）］	795	313（48.7）	90（59.2）	*χ*^2^＝5.456	0.019
既往血栓史［例（％）］	764	119（32.1）	46（31.7）	*χ*^2^＝0.010	0.921
脾左肋缘下可触及［例（％）］	750	147（24.3）	49（33.8）	*χ*^2^＝5.258	0.019
HGB［g/L，*M*（范围）］	795	195（147～252）	200（154～254）	*z*＝−2.975	0.003
RBC［×10^12^/L，*M*（范围）］	795	6.95（4.11～10.64）	7.42（5.29～10.71）	*z*＝−4.550	<0.001
HCT［％，*M*（范围）］	795	58.9（45.4～77.3）	62.2（48.4～78.3）	*z*＝−4.166	<0.001
WBC≥15×10^9^/L［例（％）］	795	212（33.0）	59（38.8）	*χ*^2^＝1.870	0.172
PLT≥450×10^9^/L［例（％）］	795	348（54.1）	56（36.8）	*χ*^2^＝14.686	<0.001
异常染色体核型［例（％）］	494	19（4.7）	5（5.7）	*χ*^2^＝0.157	0.784
JZK2V617F突变负荷［％，*M*（范围）］	594	53（1～97）	64（9～93）	*z*＝−4.098	<0.001
EPO减低［例（％）］	328	251（89.3）	44（93.6）	*χ*^2^＝0.820	0.365

注：HCT：红细胞比容；EPO：促红细胞生成素

## 讨论

既往对PV患者的研究主要集中在血栓发生高危因素探讨，对生存影响因素研究相对较少。Tefferi等[3]的研究纳入1500例PV患者，5、10年累积死亡率分别为5％、12％，与本研究结果基本一致。该研究发现年龄> 61岁、WBC>10.5×10^9^/L、PLT≥450× 10^9^/L、静脉血栓史、外周血可见不成熟粒细胞以及异常染色体核型是影响PV患者OS的独立危险因素，而存在皮肤瘙痒是OS的独立保护因素[3]。基于以上研究，2013年提出IWG-PV生存预后积分系统[3]，该生存预后积分系统中的低危、中危、高危组患者中位OS时间分别为28、19、11年。本研究结果提示IWG-PV生存预后积分系统也适用于预测中国PV患者的OS。

近年来NGS研究发现，SRSF2、IDH2、ASXL1基因突变对PV患者的OS具有显著影响，被定义为PV预后不良基因（adverse mutations）[4]。既往研究提示15％的PV患者伴有至少1个预后不良基因突变，这部分患者OS显著短于不伴有预后不良基因突变的患者（中位OS期17年对8年，*P*＝0.03）[4]。近期，研究者将SRSF2突变纳入预后积分系统，提出预测PV患者OS的新预后积分系统[5]，纳入因素包括SRSF2基因突变（赋值3分）、年龄>67岁（2分）、WBC≥15×10^9^/L（1分）、血栓史（1分），将患者分为低危组（0～1分，中位OS时间24年），中危组（2～3分，中位OS时间13.1年）和高危组（≥4分，中位OS时间3.2年）。本研究由于仅99例进行了相关检测，各基因检出有突变的患者例数很少，因此，未进行按此新积分系统分组的生存分析，有待下一步累积病例来加以验证。

本研究发现年龄≥60岁是影响PV患者OS的独立危险因素，与文献[3],[5]报道的结果一致。虽然单因素分析提示WBC≥15×10^9^/L、PLT≥450×10^9^/L对OS有显著影响，但多因素分析提示其并不是独立危险因素，与文献[3]结果不一致。此外，本研究发现确诊时染色体核型异常的患者比例仅为4.6％，低于Tefferi等[3]和Tang等[10]报道的结果（分别为12％、20％）。本研究发现异常染色体核型对OS没有显著影响，也与既往研究报道[3]不一致。造成差异的原因可能包括：本研究随访时间相对较短，随访期间死亡患者例数较少，可能会给结果带来偏倚[3], [10]；本研究纳入的患者均为初诊患者，确诊时近80％的患者进行了染色体核型检测，而Tefferi等[3]的研究中染色体核型检测患者仅占41％，Tang等[10]的研究中初诊患者仅为107例，可能存在患者选择偏倚。本研究单因素分析提示脾脏肋缘下可触及是OS的保护因素，可能与脾脏肿大患者患者诊断、治疗较早有关。

本研究进行二代测序检测的初诊PV患者中未发现SRSF2和IDH2突变阳性患者，ASXL1突变阳性患者也仅为2.0％，均低于西方国家的研究报道（ASXL1 12％，SRSF2 3％，IDH2 2％）[4]，并且本研究中ASXL1突变对OS没有显著影响。既往研究报道伴JAK2 exon12突变患者OS优于JAK2V617F突变患者（*P*＝0.02），但经年龄调整后两者OS差异也没有统计学意义（*P*＝0.1）[11]。本研究中伴JAK2 exon12突变组与JAK2V617F突变组5年累积死亡率差异没有统计学意义（*P*＝0.370）。

本研究发现PDW≥15 fl是影响PV患者OS的独立危险因素，PDW≥15 fl对IWG-PV生存预后分层中危和高危患者的OS具有显著影响，但对低危患者OS没有显著影响。PDW是反映血小板体积大小的指标，PDW增高提示血小板体积大小不等。PDW减低与骨髓衰竭、再生障碍性贫血有关，而PDW增高与骨髓增殖相关[12]–[13]。PDW增高也与炎症相关。既往研究提示巨核细胞集落刺激因子（M-CSF）、粒细胞集落刺激因子（G-CSF）及IL-6可调节巨核细胞的成熟、血小板的生产及血小板的大小[14]。PDW增高也是多种实体肿瘤预后不良因素[15]–[18]。本研究发现PDW≥15 fl组确诊时较PDW<15 fl组有更高的RBC、HGB、HCT、JAK2V617F突变负荷和脾脏肿大患者比例，提示PDW≥15 fl的患者确诊时疾病负荷更重。这些因素可能与PDW增高提示OS更差相关。

综上，本研究结果显示年龄≥60岁和PDW≥15 fl是影响我国PV患者OS的独立危险因素，IWG-PV预后积分系统也适用于中国PV患者预测OS。但本研究也具有局限性，单中心回顾性研究、患者随访时间相对较短等。因此，本研究结果尚待延长随访时间及多中心研究资料加以验证。
